# Current Research Trends in Traditional Chinese Medicine Formula: A Bibliometric Review from 2000 to 2016

**DOI:** 10.1155/2019/3961395

**Published:** 2019-03-03

**Authors:** Yi-Bing Chen, Xiao-Fang Tong, Junge Ren, Chun-Quan Yu, Yuan-Lu Cui

**Affiliations:** ^1^Tianjin University of Traditional Chinese Medicine, Tianjin 301617, China; ^2^Research Center of Traditional Chinese Medicine, Tianjin University of Traditional Chinese Medicine, Tianjin 301617, China; ^3^Key Research Laboratory of Prescription Compatibility among Components, Tianjin University of Traditional Chinese Medicine, Tianjin 301617, China; ^4^Tianjin University Library, Tianjin 300072, China

## Abstract

**Background:**

Traditional Chinese Medicine Formula (TCMF) study has been recognized widely by medical scientists around the world. However, few researchers have analyzed and summarized the rapid growth of academic articles of TCMF published in English. The primary aim of this work was to assess the outcome of these research outputs in the TCMF field from 2000 to 2016 and to evaluate the situation and tendency.

**Methods:**

Research datasets were acquired from the Web of Science database, which includes all academic articles published from 2000 to 2016; articles were tracked by the keywords “Traditional Chinese Medicine”, “Traditional Chinese Medicine Formula”, and “Chinese herb formula”. Moreover, visualization software CiteSpace V was used to analyze and generate visualization knowledge maps.

**Results:**

In total, 26,917 articles appeared in the Web of Science database, and only 2,621 publications met requirement based on reading the abstract or full text. The annual publications total, list of journals, research interests, list of medicine names, disease types, and the top 20 cited articles were given in this research paper. In addition, we compared the research of Japan and Korea TCMF, in the appendix.

**Conclusion:**

This review demonstrates that increasingly more researchers have interest in the TCMF and TCMF has great significant advantages over other areas of focus. However, these publications were published rarely in top academic journals and most best-quality papers have bias toward medical analysis rather than pharmacology. To make a breakthrough in TCMF field, further investigation is required to place emphasis on the deepening study of the mechanism of related TCMF.

## 1. Background

Advances in modern science and technology have contributed greatly to the development of the pharmaceutical industry and clinical medicine. However, East Asian countries are still generally using Traditional Chinese Medicine due to the impact on culture and history. The WHO has asserted that the Traditional Medicine is one of the primary sources of healthcare [1]. Furthermore, because of Prof. Youyou Tu's extraordinary achievement in using artemisinin treatment, she has been horned with the Physiology or Medicine Award from the Nobel Prize Organization in 2015. Hence, Traditional Chinese Medicine has attracted increasingly more attention in the global medical community [2]. In general, the Traditional Chinese Medicine Formula (TCMF) has two or more kinds of herbs as components, is designed for relatively certain symptoms, and is an important measure of the Chinese medicine treatment of diseases [3]. Knowledge regarding TCMF was not accepted in the Western Society in earlier years [4]; hence it was very hard to find the articles in peer-reviewed journals. Fortunately, the Chinese government constantly has supported the TCMF field, for example, the approval of the State Council, National Science & Technology Major Project of China “Key New Drug Creation and Manufacturing Program” launched in 2008. Thus, at a turning point, the year of 2010, the status of the Traditional Chinese Medicine has improved greatly, and the TCMF eventually has gained its deserved reputation.

Bibliometrics is a statistical analysis of written publications [5], and it is also a useful tool and method to evaluate or summarize research results in the particular field [6]. The vital indicator of estimating the quality of one article is citation rate [7]; citation analysis is necessary to provide a multidimensional summary for articles in a specific field. In addition, the TCMF research field is rich in intrinsic connection and external divergence because of its own characteristics. CiteSpace, an appropriate piece of software and a useful tool that focuses on visualizing and analyzing trends and patterns in scientific literature, was applied to conduct bibliometric analysis [8]. The results of the analysis are important for the future research of TCMF. Therefore, this paper considers bibliometrics from the perspective of article and citation analysis, which will help guide researchers or research funding agencies toward areas where there is lack of focus on research activity and provide the reader with insight and valuable information.

## 2. Methods

### 2.1. Search Strategy

Papers published from 2000 to 2016 were tracked by the keywords “Traditional Chinese Medicine”, “Traditional Chinese Medicine Formula”, and “Chinese Herb Formula” for inclusion in analysis and summarization, based on their presence in the Web of Science database. Web of Science is an online subscription-based scientific citation indexing service originally produced by the Institute for Scientific Information. It is the largest comprehensive academic information resource with the largest number of disciplines. It contains more than 12,000 core academic journals with the most influential research fields in natural sciences, engineering technology, and biomedicine [9].

During the article-collecting process, we encountered some difficulties such as “inaccurate screening result”—some formulae abbreviations or herb-pairs appear in the articles but without a detailed-explaining—and “undesired document type”—original and review articles are the only document types in this study, and meeting, editorial, and other document types are nontarget samples. Hence, these “biased articles” were not included in our finalized dataset. Therefore, the statistical analysis would be more trustworthy and accurate. The citations were gathered within one day on December 31, 2017, to avoid the possibility of unfairness due to the daily database update. The papers in this TCMF analysis were published in English in many international journals, all the authors are from China (including Mainland, Hong Kong, Macao, Taiwan), and the first author's region was determined by his/her organization regardless of citizenship and residence status.

### 2.2. Data Analysis

The papers search from the Web of Science were transferred to EndNote X7 (Thomson Reuters, San Francisco, CA) for classification and statistical analysis. Basic information such as year of publications, journals, region, and citations was recorded directly for analysis. The following information, research interest, medicine name, and disease type, was reviewed by reading the abstract or full text for statistics. Finally, the result sorting process was generated by Microsoft Office Excel® 2007. The categorical data are presented as integer and percentages. CiteSpace V was used to visualize and verify the high-frequency keywords, categories, and authors in a graphical way [10]. The parameters of CiteSpace V were as follows: time slicing (2000–2016), years per slice (1), term source (all selection), node type (choose one at a time), selection criteria (top 50), and pruning (pathfinder).

With the purpose of conducting deeper research and evaluating the international recognition of TCMF, we also screened TCMF-related articles from Japanese and South Korean publications by using the same methodology and then alphabetized the TCMF in a descending order (Z-A) according to Chinese Pinyin, Japanese Romaji, and South Korean Romaji. Please refer to the Supplementary Materials for details.

## 3. Results

**Figure 1:**
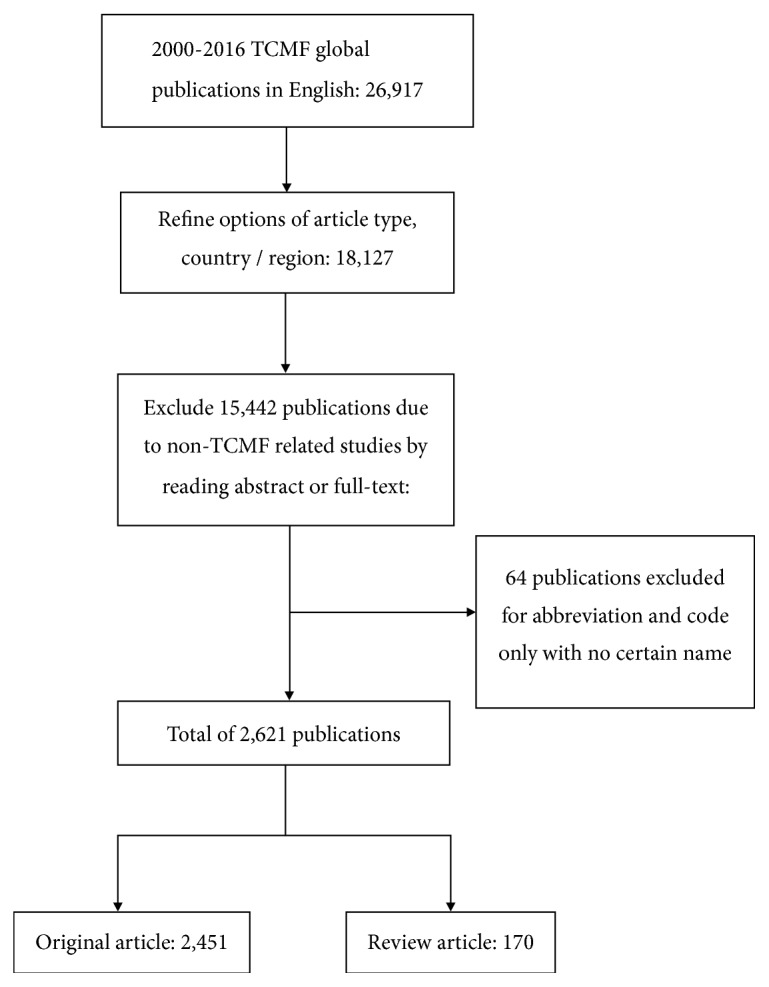
Flow chart of publications search and study selection.

A total of 26,917 TCMF-related articles were published in the period from 2000 to 2016. Refining conditions and reading the abstract or the full text aim to reduce statistical bias. As a result, a total of 2,621 articles were used from the search strategy reported above. Among these articles, 2,451 were original articles and 170 were review articles.  Figure 1 showed the search methodology and the corresponding results. Moreover, 2,233 articles were published in Mainland China, 273 were published in Taiwan, 107 were published in Hong Kong, and 8 were published in Macao.

To begin with the statistical analysis, 1,345 articles used animals as the research objects, 406 articles used cells as the research objects, and 383 used patients as the research objects. Certainly, many pharmacological studies are not only on animals but also on cells.

### 3.1. Tendency of Article Publication

The number of annual publications in 2000 to 2016 was given in Table 1. It indicated that almost 63% of articles were published during the recent four years (2013-2016). Regarding such limitations, considering that there is a language barrier and less knowledge about the Traditional Chinese Medicine, TCMF-related articles were rarely published in international journals from 2000 to 2004. Due to the Chinese government support and the research's efforts and exploration, notably, the annual publication amount has gradually increased.  Table 2 showed that there was an obvious two-sided preference between the journals and TCMF researchers; among all these TCMF publications, approximately 25% were from the* Journal of Ethnopharmacology* and* Evidence-Based Complementary and Alternative Medicine*. The articles published in these top 20 journals account for 56.1% of the total.

### 3.2. Research Content

Moreover, Table 3 showed the research interests of research production and Table 4 listed the top 20 popular disease types. Pharmacology was the trendiest research interest in the TCMF field of study; among these 2,621 papers, approximately 1,515 (57.80%) papers were pharmacology related, followed by pharmaceutical analysis (691, 26.36 %) and clinical research (381, 14.54 %). More than 300 disease types were mentioned in the datasets, and Table 4 showed that cardiovascular disease and cancer were mostly discussed. The subcategories of cardiovascular disease and cancer types are also listed in detail in Table 5.

Table 6 is a list of top 20 TCMF names, traditional herb formulae, and modern proprietary Chinese medicines, including more than 800 summarized formulae. Specifically, Huang Lian Jie Du had been studied in 62 publications, followed by Bu Yang Huan Wu Tang (49) and Shen Fu injection (44).  Table 7 shows the top 20 highly cited TCMF-related articles.

### 3.3. CiteSpace Visualization Analysis

CiteSpace V was used to generate a keyword map, category map, and coauthor map. The top 25 keywords and categories with the strongest citation burst were also drawn.

**Figure 2:**
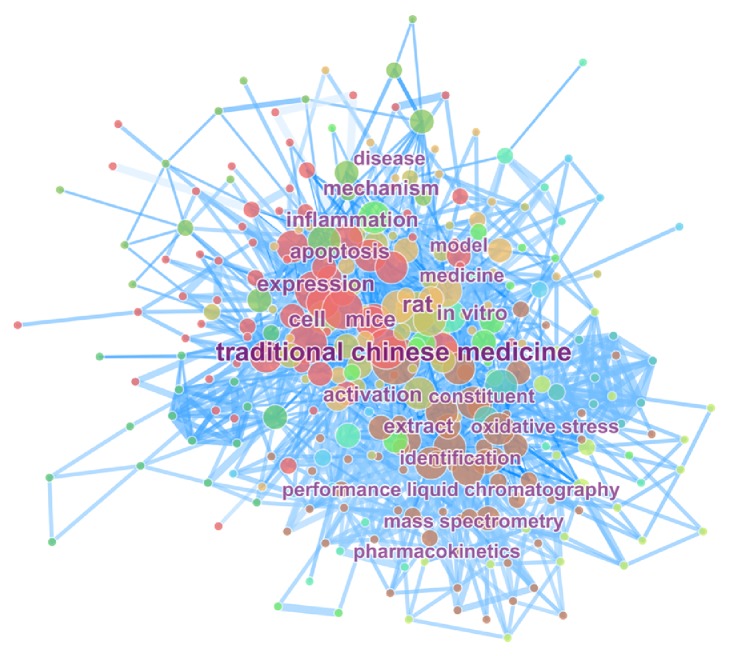
Keyword map related to TCMF research from 2000 to 2016. A total of 308 nodes and 1521 connection lines were obtained. The size and position of nodes represent research frequency and the level of core of the keyword, respectively. The darkness of blue line represents the different years.

**Figure 3:**
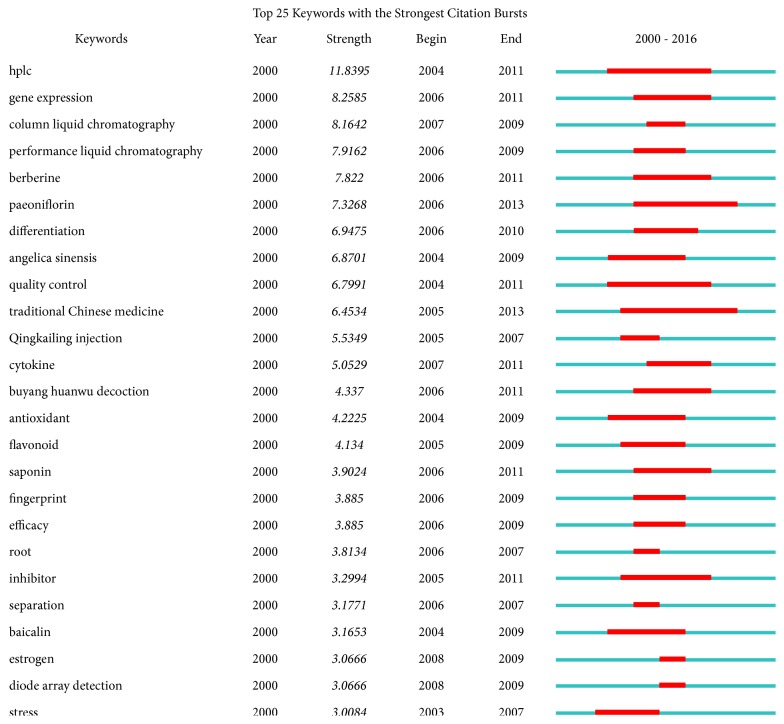
Top 25 keywords with the strongest citation burst. The red bars and blue bars demonstrate that some keywords are cited frequently and infrequently in a certain period.

Combining Figures 2 and 3, an analysis in terms of keyword counts, “HPLC” and “quality control”, indicated that the researchers may have greater research interests in drug chemical analysis and drug composition identification during 2004-2011; flavonoids, such as baicalin and berberine, and glycosides, such as paeoniflorin, were prominent from 2004 to 2011. These Traditional Chinese Medicine monomers above as natural medicine were highly recognized; their excellent antioxidation effects are also the main mechanism of many pharmacological studies [11–14].

**Figure 4:**
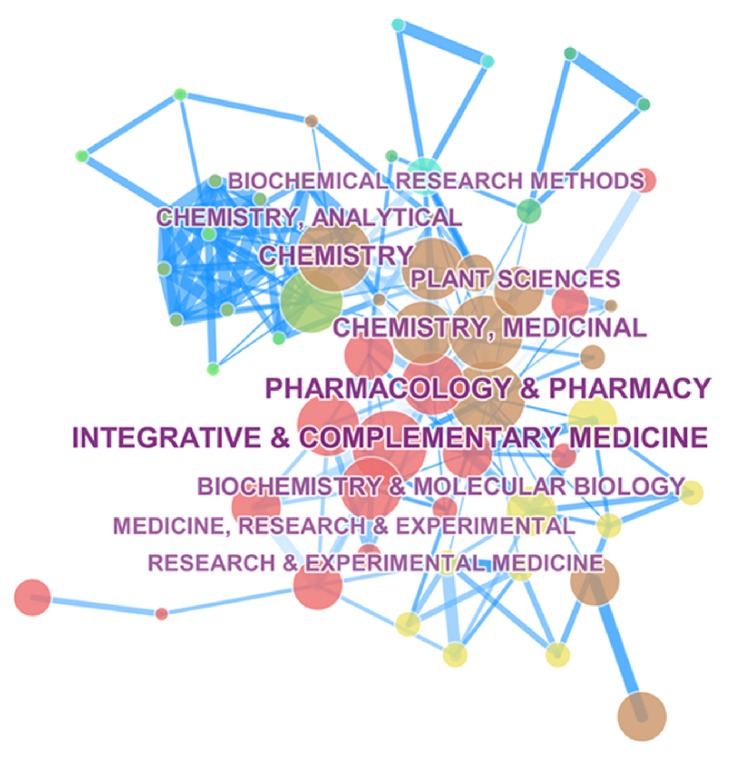
Category map related to TCMF research from 2000 to 2016. A total of 66 nodes and 194 connection lines were obtained. The size and position of nodes represent research frequency and the level of core of the category, respectively. The darkness of blue line represents the different years.

**Figure 5:**
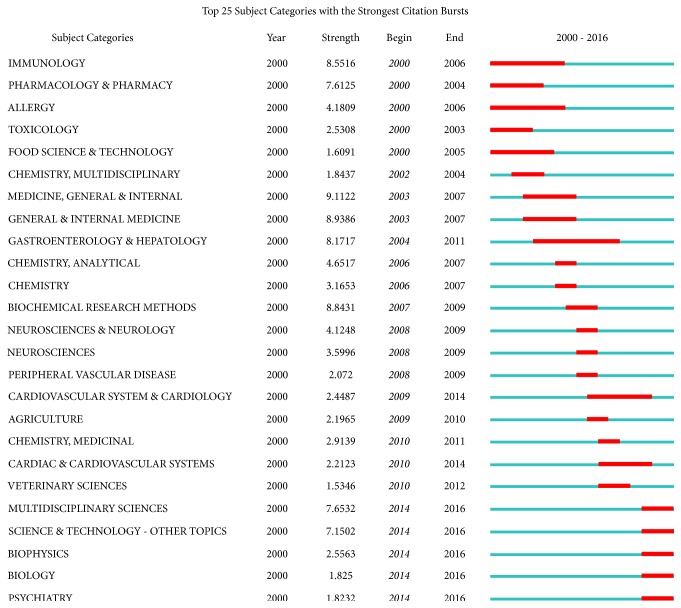
Top 25 categories with the strongest citation burst. The red bars and blue bars mean some keywords are cited frequently and infrequently in a certain period.

Category could help researchers understand the focus of TCMF research, combining Figures 4 and 5, immunology, pharmacology, and drug analysis were featured in the early stage, but research on the cardiovascular system was in-depth from 2009 to 2014, and the focus of research from 2014 to 2016 shifted to biophysics, science and technology, and other multidisciplinary sciences.

**Figure 6:**
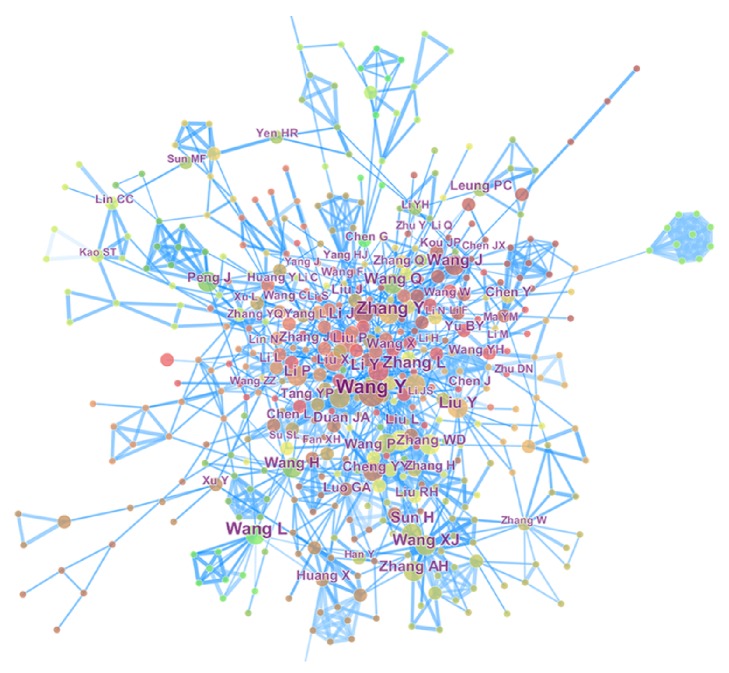
Coauthor map related to TCMF research from 2000 to 2016. A total of 536 nodes and 1621 connection lines were obtained. The size and position of nodes represent research frequency and the level of core of the author, respectively. The distance and connection of nodes represent the author's cooperation. The darkness of blue line represents the different years.

Figure 6 shows the coauthor map; this analysis result revealed the cooperative relationship between the authors. Coauthor analysis can be used as an important tool in the evaluation of bibliometrics to identify research groups in “unknown” universities or organizations [15].

## 4. Discussion

This report analyzed TCMF-related articles published in international journals from 2000 to 2016. To our knowledge, this is the first report that analyzed the quality and quantity of TCMF-related studies in China. More than 26,000 articles were obtained when using “Traditional Chinese Medicine”, “Traditional Chinese Medicine Formula”, and “Chinese herb formula” as the keywords in a search of the Web of Science database. A total of 2,621 articles were screened out by reading the abstract or full text. Each database has its own advantages and characteristics. A possible explanation for choosing the Web of Science as the only database is that it offered several advantages: First of all, Web of Science, with high recognition and article quality for many years, offers a powerful analysis of data from various aspects and the selected articles. Second, Web of Science has a function of EndNote online. During the items collecting process, the documents that meet the requirements could be added to the EndNote online at any time and there was logo displayed on the web pages. This unique feature could easily transfer the articles to the EndNote software in order to conduct the future analysis. Third, every database has its own standard on calculating the citation rate of article and evaluating the importance and influence of specific publications. Hence, choosing Web of Science could guarantee a powerful uniform citation rate and impact of journals.

Chinese medicine culture has existed in China for thousands of years, since the era of ancient China; countless research studies have been written by Chinese medicine scholars. However, due to the limited ability of research and the language barrier, TCMF publications were not favored in the international journals. Fortunately, the amount of TCMF publications has increased remarkably over time, especially after 2010. With support from Chinese government and the development of science and technology, we do observe increasingly more scientific research outputs from the medicine community. A significant contribution to the TCMF development occurred when Prof. Youyou Tu won the Physiology or Medicine Noble Prize [2], which encourages Traditional Chinese Medicine professionals to explore more about the TCMF research contents. Thus, a breakthrough in the TCMF field of research is the key to a brighter future for the medicine community.

According to the preparatory work, TCMF-related articles have been published in more than 300 journals. Most of the TCFM articles were published in the* Journal of Ethnopharmacology *and* Evidence-Based Complementary and Alternative Medicine*, which matches the result from Sa'ed H. Zyoud's bibliometric analysis of the Integrative and Complementary Medicine field [16]. This means that the* Journal of Ethnopharmacology *and* Evidence-Based Complementary and Alternative Medicine *are more likely to accept Traditional Medicine articles, when compared with the other publications.

Furthermore, pharmacology study has been recorded as the most popular research filed; 1,515 out of 2,621 articles discussed pharmacology. Pharmaceutical analysis is the second favorable topic with 691 out of 2.621 articles; 381 out of 2,621 articles had a clinical research focus. In pharmaceutical analysis study, the top three trendy topics are metabolomics, pharmacokinetics, and quality control. As reported in our database, there are 173, 167 and 82 articles conducted on these three areas, respectively. Fifty-one pharmacological analysis articles were included for the clinical study. The secondary major research concentration on clinical study was meta-analysis; 54 related articles were included. We have listed all the TCMF research topics that were being discussed widely. We hope this information will be helpful for future study.

Many of diseases could be cured through the TCMF treatment. For instance, approximately, 300 diseases that use TCMF methodology for treatments were mentioned in screening publications. Furthermore, studies on cardiovascular disease and cancer are mostly being analyzed, followed by diabetes and depression. A possible reason for this would be that cardiovascular disease and cancer are the most fatal diseases [17–19]. With the increasing pressure of modern life and the irregular lifestyles, diabetes and depression have become increasingly threatening to human health [20–23].

Countless Traditional Chinese Medicine and Chinese patent medicines are in the historical records; meanwhile, these medicines are also being used widely in the market. As noted, more than 800 types of Traditional Chinese Medicine were included in the database. The reason that Huang Lian Jie Du Tang was mentioned broadly is that the major three components of this medicine,* Coptis chinensis Franch, Scutellaria baicalensis Georgi, *and* Phellodendron chinense Schneid*, all contain flavonoid and alkaloid. Flavonoid and alkaloid work well for an anti-inflammatory effect [24, 25]. Huang Lian Jie Du Tang is an effective treatment for inflammation [26], and inflammatory actions lead to many disorders [27–29]. Tonic formulae, such as Sheng Mai san, Liu Wei Di Huang Wan, and Si Wu Tang, and blood-regulating formulae, such as Bu Yang Huan Wu tang and Xue Fu Zhu Yu tang, were listed in Table 6 as the top 20 most popular TCMF, and most of these formulae could be used in cardiovascular and cancer treatments [30–34]. Moreover, other formulae such as Kai Xin San and Xiao Yao San are commonly used for mental illness treatment [35, 36], and Dang Gui Bu Xue Tang and Huang Lian Jie Du Tang could also be adopted for diabetes treatment [37, 38]. Thus, from these findings we can establish a clear mutual relationship between the tendency of TCMF studies and common diseases.

From Table 7, we can see that pharmaceutical analysis was the most frequently cited term in TCMF articles; this article previously mentioned that pharmacology is the most favorable research topic in TCMF study. Indeed, some respected journals such as* Analytica Chimica Acta, Chromatography A*, and* Analyst *are subject to the Chemistry section in the Science Citation Index. This evidence illuminates a strong argument that pharmaceutical analysis subject can be highly transferable and widely adopted in other fields. At the same time, some formulae were discussed frequently by TCMF scholars such as Huang Lian Jie Du Tang, Si Wu Tang, Dang Gui Bu Xue Tang, and Liu Wei Di Huang Wan; these are all included in the highly cited papers, as we previously mentioned. It should be mentioned that the Yin Chen Hao Tang was not included in Table 6, but there were still two highly cited articles that gave a detailed analysis of the formula. There are five pharmacological articles listed in Table 7; among them, one article is using Compound Dan Shen Formula in cardiovascular disease, and two articles are the application of Ban Xia Hou Po Tang in depression treatments. These were also diseases garnering a high level of attention. The above analysis demonstrated that research content and research interest, compound name, and disease type of TCMF were inseparable, which can provide a comprehensive understanding of the current development of TCMF.

Knowledge domain visualization, and visualization of scientific literature can strengthen the scientific and objective nature of bibliometrics and meet more practical needs, such as helping researchers analyze potential knowledge contained in scientific analysis [39, 40]. In this paper, the bibliometric research of TCMF placed a particular emphasis on the statistics of research content and quantity. The subjective analysis in the work could be supplemented with the CiteSpace V software, and the literature bibliometric research of TCMF could offer more in-depth and comprehensive information.

There are many articles on the research of the TCMF, whether in Asian countries or Western countries, but our work mainly analyzes the published trend of the articles on TCMF in China and concerned part of other Asian countries in recent years, which does not involve Western countries, because China is in the majority in leading this research so far. Moreover, most of the research on TCMF has been published in Chinese, and Chinese papers were not included in this study. However, according to the scientific research management orientation in recent years in China, excellent research results have been published in English journals. Therefore, this study has included the most important research which is representative of TCMF. Nevertheless, we still hope to see that more and more domestic and foreign scholars can pay attention to the study of TCMF.

From 2013 to 2016, TCMF study was being improved greatly by the Traditional Chinese Medicine researchers. For instance, the TCMF articles published in this period account for 63% of the total article amount. This scenario indicates that increasingly more scholars have interest in TCMF study. Further investigation is required to focus on the treatment of certain diseases such as cardiovascular disease and cancers. A deeper understanding and knowledge of each Traditional Chinese Medicine's component is essential because of the complexity and particularity of TCMF. Moreover, cooperating and communicating with scholars from other countries would boost the development of TCMF study.

## 5. Conclusion

In summary, for future TCMF studies, we hope future scholars could diversify the research contents, extend the horizon of the cognitive perspectives of Traditional Chinese Medicine, and deepen the professional knowledge of TCMF research. For Traditional Chinese Medicine scholars, it is inappropriate to use the outcomes of treatment as the only standard to evaluate the effectiveness of TCMF. To make the breakthrough, the priority is to study the treatment mechanism of the disease on the selected formula and to clarify the effective components of the formula. This would enhance the authority and accuracy of TCMF study and help remove the stereotypes of Traditional Chinese Medicine as being “unscientific” and “empiricist”. We also hope that with the improvement of the research level of TCMF, more best-quality papers will be published in important academic journals such as* Nature *and* Science*.

## Figures and Tables

**Table 1:** The number of annual publications of Traditional Chinese Medicine Formula related articles from 2000 to 2016.

Year	Number	Percentage( /2621)

2016	478	18.23%
2015	450	17.17%
2014	380	14.50%
2013	344	13.12%
2012	212	8.09%
2011	180	6.87%
2010	128	4.88%
2009	104	3.97%
2008	92	3.51%
2007	67	2.56%
2006	63	2.40%
2005	42	1.60%
2004	24	0.92%
2003	23	0.88%
2002	12	0.46%
2001	9	0.34%
2000	13	0.50%

**Table 2:** List of top 20 journals in which articles were published and the newest impact factor (IF)^∗^.

Rating	Journal	Number	IF

1	Journal of Ethnopharmacology	379	3.115
2	Evidence-Based Complementary and	247	2.064
	Alternative Medicine		
3	Journal of Pharmaceutical and	99	2.831
	Biomedical Analysis		
4	BMC Complementary and Alternative	78	2.109
	Medicine		
5	PLOS One	66	2.766
6	Journal of Separation Science	56	2.415
7	Chinese Journal of Integrative Medicine	55	1.346
8	Biomedical Chromatography	53	1.688
9	American Journal of Chinese Medicine	50	3.120
10	Journal of Traditional Chinese Medicine	50	0.857
11	Journal of Chromatography B-Analytical	45	2.441
	Technologies in the Biomedical and Life		
	Sciences		
12	Neural Regeneration Research	42	2.234
13	Scientific Reports	40	4.122
14	Experimental and Therapeutic Medicine	35	1.410
15	Molecules	37	3.098
16	Chinese Medical Journal	32	1.596
17	Molecular Medicine Reports	30	1.922
18	Trials	27	2.067
19	Analytical Methods	26	2.073
20	Chromatographia	25	1.401

∗The impact factor was reported according to Institute for Scientific Information (ISI) journal citation reports (JCR) 2018.

**Table 3:** Research interests of articles by reading abstracts or full-texts.

Research interests	Number (%)∗

Pharmacology	1515 (57.80%)
Pharmaceutical analysis	691 (26.36%)
Clinical research	381 (14.54%)
Mathematical Analysis and System Biology	64 (2.44)
Toxicology	32 (1.22%)
Pharmaceutical preparations	27 (1.03%)
Pathology	4 (0.02%)

∗Total percentage exceeds 100% due to multidiscipline interaction.

**Table 4:** List of the top 20 types of disease appearing in articles

Rating	Disease types	Number

1	Cardiovascular disease	335
2	Cancer	156
3	Diabetes	105
4	Depression	108
5	Alzheimer's disease	48
6	Asthma	40
7	Liver fibrosis (hepatic fibrosis)	40
8	Rheumatoid arthritis	33
9	Osteoporosis	32
10	Liver injury	26
11	Dementia	26
12	Diabetic nephropathy	24
13	Chronic obstructive pulmonary disease	23
14	Osteoarthritis	22
15	Insomnia	22
16	Ulcerative colitis	20
17	Fatigue	19
18	Parkinson's disease	18
19	Headache	18
20	Dysmenorrhoea	17

**Table 5:** The top types of cardiovascular diseases and cancers.

Cardiovascular disease	Number	Cancer	Number

Ischemic stroke	37	Anti-tumor	33
Hypertension	33	Breast cancer	25
Myocardial infarction	29	Colorectal cancer	21
Heart failure	29	Hepatocellular carcinoma	14
Stroke	29	Lung cancer	13
Atherosclerosis	24	Gastric cancer	12
Chronic heart failure	14	Liver cancer	10
Sepsis	14	Bone cancer	6
Coronary heart disease	13	Leukemia	4
Cardiac arrest	12	Colon cancer	4
Angina	10		

**Table 6:** The top 20 popular Traditional Chinese Medicine Formulas involved in articles.

Rating	Traditional Chinese Medicine Formula	Number

1	Huang Lian Jie Du tang	62
2	Bu Yang Huan Wu tang	49
3	Shen Fu injection	44
4	Sheng Mai san	44
5	Liu Wei Di Huang wan	44
6	Si Wu tang	42
7	Da Cheng Qi tang	41
8	Dang Gui Bu Xue tang	37
9	Tong Xin Luo capsule	32
10	Si Ni san	30
11	Xue Fu Zhu Yu tang	29
12	Kai Xin san	29
13	Xiao Chai Hu tang	27
14	Xiao Yao san	25
15	Xie Xin tang	25
16	Pien Tze Huang	24
17	Ge Gen Qin Lian tang	22
18	Jia Wei Xiao Yao San	20
19	Bu Zhong Yi Qi tang	20
20	Dan Hong injection	19

**Table 7:** The top 20 highly cited Traditional Chinese Medicine Formula-related articles.

Rating	Title	Journal	Author &Year	Cited Time

1	Multiple chromatographic fingerprinting and its application to the quality control of herbal medicines	*Analytica Chimica Acta*	Fan, X. H. 2006	128
2	Comparative pharmacokinetics of baicalin after oral administration of pure baicalin, Radix scutellariae extract and Huang-Lian-Jie-Du-Tang to rats	*Journal of Ethnopharmacology*	Lu, T. 2007	119
3	Analysis of the constituents in the rat plasma after oral administration of Yin Chen Hao Tang by UPLC/Q-TOF-MS/MS	*Journal of Pharmaceutical and Biomedical Analysis*	Wang, X. J. 2008	113
4	Influences of traditional Chinese medicine on non-specific immunity of Jian Carp (Cyprinus carpio var. Jian)	*Fish & Shellfish Immunology*	Jian, J 2004	114
5	Herb network construction and co-module analysis for uncovering the combination rule of traditional Chinese herbal formulae	*BMC Bioinformatic*	Li, S. 2010	105
6	An approach to develop two-dimensional fingerprint for the quality control of Qingkailing injection by high-performance liquid chromatography with diode array detection	*Journal of Chromatography A*	Yan, S. K 2005	98
7	Chemical and biological assessment of a Chinese herbal decoction containing radix astragali and radix angelicae sinensis: Determination of drug ratio in having optimized properties	*Journal of Agriculture and Food Chemistry *	Dong, T. T. X. 2006	96
8	Chemical and biological assessment of a traditional Chinese herbal decoction prepared from Radix Astragali and Radix Angelicae Sinensis: Orthogonal array design to optimize the extraction of chemical constituents	*Planta Medica*	Song, Z. H. 2004	82
9	Efficacy and tolerability of a Chinese herbal medicine concoction for treatment of atopic dermatitis: a randomized, double-blind, placebo-controlled study	*British Journal of Dermatology*	Hon, K. L. E. 2007	82
10	Chinese herbal formulas for treating hypertension in traditional Chinese medicine: perspective of modern science	*Hypertension Research*	Xiong, X. J. 2013	77
11	Pharmacokinetics screening for multi-components absorbed in the rat plasma after oral administration traditional Chinese medicine formula Yin-Chen-Hao-Tang by ultra performance liquid chromatography-electrospray ionization/quadrupole-time-of-flight mass spectrometry combined with pattern recognition methods	*Analyst*	Wang. X. 2011	76
12	Metabonomic Study of Chinese Medicine Shuanglong Formula as an Effective Treatment for Myocardial Infarction in Rats	*Journal of Proteome Research*	Liang, X. P. 2011	75
13	Simultaneous determination of berberine and palmatine in rat plasma by HPLC-ESI-MS after oral administration of traditional Chinese medicinal preparation Huang-Lian-Jie-Du decoction and the pharmacokinetic application of the method	*Journal of Pharmaceutical and Biomedical Analysis*	Lu, T. 2006	72
14	Antidepressant effects of Banxia Houpu decoction, a traditional Chinese medicinal empirical formula	*Journal of Ethnopharmacology*	Luo, L 2000	71
15	Identification and determination of the major constituents in traditional Chinese medicine Si-Wu-Tang by HPLC coupled with DAD and ESI-MS	*Journal of Pharmaceutical and Biomedical Analysis*	Zhang, H. J. 2004	68
16	A System-Level Investigation into the Mechanisms of Chinese Traditional Medicine: Compound Danshen Formula for Cardiovascular Disease Treatment	*PLOS ONE*	Liu, X. X. 2012	68
17	Behavioral and biochemical studies on chronic mild stress models in rats treated with a Chinese traditional prescription Banxia-houpu decoction	*Life Science*	Li, J. M. 2003	68
18	Identification and determination of the major constituents in Traditional Chinese Medicinal formula Danggui-Shaoyao-San by HPLC-DAD-ESI-MS/MS	*Journal of Pharmaceutical and Biomedical Analysis*	Chen, L. L. 2009	67
19	Anti-inflammatory effects of Huang-Lian-Jie-Du decoction, its two fractions and four typical compounds	*Journal of Ethnopharmacology *	Lu, J. 2011	67
20	Rapid and sensitive screening and characterization of phenolic acids, phthalides, saponins and isoflavonoids in Danggui Buxue Tang by rapid resolution liquid chromatography/diode-array detection coupled with time-of-flight mass spectrometry	*Rapid Communications in Mass Spectrometry*	Qi, L. W. 2008	67

## Supplementary Materials

Supplementary Materials Japanese and South Korean research on Traditional Chinese Medicine Formula were compared with Chinese research, listing more than 60 kinds of Traditional Chinese Medicine Formulae in Roman alphabet match table according to Chinese Pinyin, Japanese Romaji, and South Korean Romaji to facilitate the exchange among researchers in the above three countries.
