# Supplementary material for: Current Research Trends in Traditional Chinese Medicine Formula: A Bibliometric Review from 2000 to 2016
Source: Evid Based Complement Alternat Med. 2019 Mar 3;2019:3961395. doi: 10.1155/2019/3961395 (PMC6420998; doi:10.1155/2019/3961395)
Supplement: Supplementary Materials — Japanese and South Korean research on Traditional Chinese Medicine Formula were compared with Chinese research, listing more than 60 kinds of Traditional Chinese Medicine Formulae in Roman alphabet match table according to Chinese Pinyin, Japanese Romaji, and South Korean Romaji to facilitate the exchange among researchers in the above three countries. [file 3961395.f1.docx]

**Supplementary material**

This part demonstrated the current TCMF research situation in other countries by listing a detailed comparison, which is an analysis of Japanese and South Korean TCMF related publications. China has a long history of influencing the other countries on Traditional Chinese Medicine study. The countries that in which TCMF has been introduced systematically and comprehensively to are Japan and South Korea [1]. Even though Japan and South Korean have followed the Chinese fundamental theoretical system on Traditional Medicine study for the most recent hundreds of years, these two countries have their own research focuses, which are not the same as those in China. For instances, Yi Gan San or Yokukasan [2-5] in Japanese Romaji is the most popular TCMF research area, and Shi Quan Da Bu Tang or Sipjeondaebotang [6-8]. Medicine as the mainstream medicine is accepted and utilized broadly by the majority in Japan [9]. In addition, such limitations on Traditional Chinese Medicine approval and import make it even harder to spread TCMF knowledge in Japan. Compared with Japan, South Korea is relatively active in Traditional Chinese Medicine study, and it also has integrated its own cultural elements into Traditional Chinese Medicine theory, to build their unique medical system [10]. Therefore, approximately 60 kinds of formulae were listed in Table S1 with a descending alphabetical order according to Chinese Pinyin, Japanese Romaji and South Korean Romaji.

**Reference**

1. Xue YM. Chinese medicine introduced to Japan and South Korea. J Tradit Chin

Med Lit. 2009; 27(4): 47-48.

2. Matsumoto T, Kushida H, Maruyama T, Nishimura H, Watanabe J, Maemura K,

Kase Y. In vitro identification of human cytochrome P450 isoforms involved in the metabolism of Geissoschizine methyl ether, an active component of the traditional Japanese medicine Yokukansan. Xenobiotica. 2016; 46(4): 325-334.

3. Ogawa Y, Fujii Y, Sugiyama R, Konishi T. The role of the seven crude drug

components in the sleep-promoting effect of Yokukansan. J Ethnopharmacol. 2016; 177: 19-27.

4. Uchida N, Egashira N, Iwasaki K, Ishibashi A, Tashiro R, Nogami A, Manome N,

Abe M, Takasaki K, Mishima K, Takata J, Oishi R, Nishimura R, Fujiwara M. Yokukansan Inhibits Social Isolation-Induced Aggression and Methamphetamine-Induced Hyperlocomotion in Rodents. Biol Pharm Bull. 2009; 32(3): 372-375.

5. Kawanabe T, Yoritaka A, Shimura H, Oizumi H, Tanaka S, Hattori N. Successful

treatment with Yokukansan for behavioral and psychological symptoms of Parkinsonian dementia. Prog Neuro-Psychoph. 2010; 34(2): 284-287.

6. Park HR, Lee H, Park H, Cho WK, Ma JY. Fermented Sipjeondaebo-tang

Alleviates Memory Deficits and Loss of Hippocampal Neurogenesis in Scopolamine-induced Amnesia in Mice. Sci Rep. 2016; 6: 22405

7. Shin IS, Yu YB, Seo CS, Ha HK, Lee MY, Huang DS, Kim JH, Shin HK.

Subchronic toxicity of Sipjeondaebo-tang (SDT) in Sprague-Dawley rats. Regul Toxicol Pharm. 2011; 59(3): 375-384.

8. Choi YK, Jung KY, Woo S-M, Yun YJ, Jun CY, Park JH, Shin YC, Cho SG, Ko SG.

Effect of Sipjeondaebo-Tang on Cancer-Induced Anorexia and Cachexia in CT-26 Tumor-Bearing Mice. Mediat. Inflamm. 2015; 2014(2015), 736563..

9. Saijirahu. The development of traditional Chinese medicine in modren Japan. Int J

Tradit Chin Med. 2015; (7): 583-589.

10. Yuan WL, Meng JY, Tai LZ, Jin J. Current situation of South Korean Medicine in

South Korea Daejeon university. World J.Integrated Tradit Western Med. 2011; 06(8): 719-721.

Table S1 The list of descending alphabetical order (Z-A) according to Chinese Pinyin, Japanese Romaji and South Korean Romaji

| **Chinese** | **Pinyin** | **Japanese romaji** | **Korean romaji** |
| --- | --- | --- | --- |
| 滋阴降火汤 | ziyinjianghuotang | jininkoukato | jaeumganghwatang |
| 栀子厚朴汤 | zhizihoupotang | N/A | chijahubaktang |
| 炙甘草汤 | zhigancaotang | shakanzoto | jakamchotang |
| 真武汤 | zhenwutang | shinbuto | jinmutang |
| 玉屏风散 | yupingfengsan | gyokuheifusan | okbyungpoongsan |
| 越鞠丸 | yuejuwan | N/A | wolgukhwan |
| 茵陈蒿汤 | yinchenhaotang | inchinkoto | yinjinhotang |
| 抑肝散 | yigansan | yokukansan | yeokgansan |
| 辛夷清肺汤 | xinyiqingfeitang | shiniseihaito | N/A |
| 小青龙汤 | xiaoqinglongtang | shoseiryuto | socheogryongtang |
| 小柴胡汤 | xiaochaihutang | shosaikoto | soshihotang |
| 香砂平胃散 | xiangshapingweisan | koushaheiisan | hyangsapyungwisan |
| 吴茱萸汤 | wuzhuyutang | goshuyuto | osuyutang |
| 五苓散 | wulingsan | goreisan | oryeonggan |
| 调胃承气汤 | tiaoweichengqitang | choijyokito | jowiseunggitang |
| 桃红四物汤 | taohongsiwutang | N/A | dohongsamultang |
| 酸枣仁汤 | suanzaorentang | sansouninto | sanjointang |
| 四物汤 | siwutang | shimotsuto | samultang |
| 四逆散 | sinisan | shigyakusan | sayeuksan |
| 四君子汤 | sijunzitang | shikunshito | sagunjatang |
| 疏经活血汤 | shujinghuoxuetang | sokeikakketsuto | sogyeonghwalhyeltang |
| 十全大补汤 | shiquandabutang | jyuzentaihoto | sipjeondaebotang |
| 参苏饮 | shensuyin | jinsoin | samsoeum |
| 参苓白术散 | shenlingbaizhusan | jinryobyakujyutsusan | samyongbakchulsan |
| 生脉散 | shengmaisan | N/A | saengmaeksan |
| 升麻葛根汤 | shengmagegentang | shomakakkonto | sungmagalkuntang |

Table S1 (continued)

| **Chinese** | **Pinyin** | **Japanese romaji** | **Korean romaji** |
| --- | --- | --- | --- |
| 芍药甘草汤 | shaoyaogancaotang | shakuyakukanzoto | jakyakgamchotang |
| 少府逐瘀汤 | shaofuzhuyutang | N/A | sobokchugeotang |
| 三黄汤 | sanhuangtang | sanouto | samhwangtang |
| 人参养荣汤 | renshenyangrongtang | ninjinyoeito | insamyangyeongtang |
| 人参败毒散 | renshenbaidusan | N/A | insampaedoksan |
| 牛车肾气丸 | niucheshenqiwan | goshajinkigan | uchasingihwan |
| 麻杏石甘汤 | maxingshigantang | makyosekikanto | N/A |
| 麦门冬汤 | maimendongtang | bakumondoto | maekmoondongtang |
| 理中汤 | lizhongtang | N/A | ijungtang |
| 六味地黄丸 | liuweidihuangwan | rokumijiogan | yukmijihwanghwan |
| 六君子汤 | liujunzitang | [rikkunshito](http://kampo.ca/herbs-formulas/formulas/rikkunshito/) | yukgunjatang |
| 九味羌活汤 | jiuweiqianghuotang | N/A | gumiganghwaltang |
| 加味逍遥散 | jiaweixiaoyaosan | kamishoyosan | N/A |
| 加味温胆汤 | jiaweiwendantang | kamiuntanto | kamiondantang |
| 藿香正气散 | huoxiangzhengqisan | N/A | gwakhyangjeonggisan |
| 黄芪建中汤 | huangqijianzhongtang | ogikenchuto | hwanggigunjungtang |
| 黄连解毒汤 | huanglianjiedutang | orengedokuto | hwangryunhaedoktang |
| 桂枝茯苓丸 | guizhifulingwan | keishibukuryogan | gyejibokryeonghwan |
| 归脾汤 | guipitang | kihito | kwibitang |
| 钩藤散 | goutengsan | chotosan | N/A |
| 葛根汤 | gegentang | kakonto | galkuntang |
| 甘麦大枣汤 | ganmaidazaotang | kanbakutaisoto | kammaegdaejotang |
| 甘草泻心汤 | gancaoxiexintang | kanzoshashinto | gamchosasimtang |
| 防风通圣散 | fangfengtongshengsan | bofutsushosan | N/A |
| 二仙汤 | erxiantang | nisento | yiseontang |
| 二陈汤 | erchentang | nichinto | ljintang |
| 当归芍药散 | dangguishaoyaosan | tokishakuyakusan | dangguijakyaksan |

Table S1 (continued)

| **Chinese** | **Pinyin** | **Japanese romaji** | **Korean romaji** |
| --- | --- | --- | --- |
| 当归补血汤 | dangguibuxuetang | tokihoketsuto | dangguibohyultang |
| 大建中汤 | dajianzhongtang | daikenchuto | daegeonjungtang |
| 大黄牡丹汤 | dahuangmudantang | daiobotanpito | N/A |
| 大黄甘草汤 | dahuanggancaotang | daiokanzoto | daehwanggamchotang |
| 大承气汤 | dachengqitang | daijyokito | daeseungkitang |
| 川芎茶调散 | chuangxiongchatiaosan | senkyuchachosan | cheongungdajosan |
| 柴朴汤 | chaipotang | saibokuto | N/A |
| 补中益气汤 | buzhongyiqitang | hochuekkito | bojungikkitang |
| 半夏厚朴汤 | banxiaohoupotang | hangekokubokuto | banhahubaktang |
